# Tryptophan hydroxylase 1 and 5-HT_7_ receptor preferentially expressed in triple-negative breast cancer promote cancer progression through autocrine serotonin signaling

**DOI:** 10.1186/s12943-016-0559-6

**Published:** 2016-11-21

**Authors:** Jaya Gautam, Suhrid Banskota, Sushil Chandra Regmi, Subi Ahn, Yong Hyun Jeon, Hyunyoung Jeong, Seung Joo Kim, Tae-gyu Nam, Byeong-Seon Jeong, Jung-Ae Kim

**Affiliations:** 1 College of Pharmacy, Yeungnam University, 280 Daehak-Ro, Gyeongsan, 38541 Republic of Korea; 2 Department of Nuclear Medicine, Kyungpook National University, Daegu, 41944 Republic of Korea; 3 Departments of Pharmacy Practice and Biopharmaceutical Sciences, College of Pharmacy, University of Illinois at Chicago, Chicago, IL 60612 USA; 4 Department of Pharmacy, Hanyang University, Ansan, 15588 Republic of Korea

**Keywords:** Triple negative breast cancer, Autocrine 5-HT action, Tryptophan hydroxylase 1, 5-HT_7_ receptor, 6-Amino-2,4,5-trimethylpyridin-3-ol

## Abstract

**Background:**

Triple-negative breast cancer (TNBC) has a high risk of relapse and there are few chemotherapy options. Although 5-hydroxytryptamine (5-HT, serotonin) signaling pathways have been suggested as potential targets for anti-cancer drug development, the mechanism responsible for the action of 5-HT in TNBC remains unknown.

**Methods:**

Quantitative real-time polymerase chain reaction (qRT-PCR) and Western blotting were used to measure mRNA and protein levels, respectively. Cell proliferation was measured using CellTiter 96 Aqueous One Solution. siRNA transfection was used to assess involvement of genes in cancer invasion, which were identified by Matrigel transwell invasion assay. Levels of 5-HT and vascular endothelial growth factor (VEGF) were measured using ELISA kits. Chick chorioallantoic membrane (CAM) assay and mouse tumor model were used to investigate the in vivo effects of SB269970, a 5-HT_7_ receptor antagonist, and BJ-1113, a novel synthetic compound.

**Results:**

TNBC cell lines (MDA-MB-231, HCC-1395, and Hs578T) expressed higher levels of tryptophan hydroxylase 1 (TPH1) than hormone-responsive breast cancer cell lines (MCF-7 and T47D). In MDA-MB-231 cells, 5-HT promoted invasion and proliferation via 5-HT_7_ receptor, and interestingly, the stimulatory effect of 5-HT on MDA-MB-231 cell invasion was stronger than its effect on proliferation. Likewise, downstream signaling pathways of 5-HT_7_ differed during invasion and proliferation, that is, Gα-activated cAMP and Gβγ-activated kinase signaling during invasion, and Gβγ-activated PI3K/Akt signaling during proliferation. Also, 5-HT increased the protein expressions of TPH1 and VEGF in MDA-MB-231 cells. These results provide insight of the stimulatory effect of 5-HT on breast cancer progression; 5-HT was found to act more strongly during the first stage of metastasis (during invasion and migration) than during the later proliferative phase after local invasion. Interestingly, these actions of 5-HT were inhibited by BJ-1113, a 6-amino-2,4,5-trimethylpyridin-3-ol analog. BJ-1113 blocked intracellular signaling pathways initiated by 5-HT_7_ receptor activation, and exhibited anti-proliferative and anti-invasive activities against MDA-MB-231 cells. Furthermore, the inhibitory effect of BJ-1113 against MDA-MB-231 tumor growth was greater than that of SB269970, a 5-HT_7_ receptor antagonist.

**Conclusions:**

5-HT_7_ receptor which mediates 5-HT-induced cancer progression is a potential therapeutic target in TNBC, and BJ-1113 offers a novel scaffold for the development of anti-cancer agents against TNBC.

## Background

Breast cancer is the most common cancer in women and the second leading cause of cancer death in women [1]. Treatment typically involves combinations of surgery, radiation therapy, and chemotherapy. Chemotherapeutic options for breast cancers depend on the expressions of estrogen receptor (ER) and progesterone receptor (PR), and on the amplification of human epidermal growth factor receptor 2 (HER-2/Neu) [2]. Triple-negative breast cancer (TNBC) does not express ER, PR, or HER-2/Neu, and is a highly aggressive subtype of breast cancer. Although TNBC is highly susceptible to chemotherapy (e.g., platinum-based agents), the risk of relapse is high because the presence of residual cancer cells is common after treatment [3–5], and these cells tend to exhibit an increased capacity for proliferation, invasion, and metastasis [6–8]. Furthermore, patients with recurrent TNBC after standard chemotherapeutic regimens have limited chemotherapeutic options [9]. Accordingly, novel molecular targets and agents for such targets are needed to treat TNBC.

A recent study demonstrated 5-HT (5-hydroxytryptamine, serotonin) production in patients with breast cancer [10], which suggests components of the 5-HT signaling pathway may offer potential drug targets. 5-HT is a neurotransmitter produced in brain. It is also produced by enterochromaffin cells in gut, which accounts for ~95% of 5-HT production, and epithelial cells in mammary glands. 5-HT is involved in multiple biological functions, such as, immune system [11], gut physiology [12], and epithelial homeostasis of various organs, such as, mammary glands, lungs, pancreas, liver, and prostate [13–17]. In the mammary gland, 5-HT produced by the prolactin-induced expression of the 5-HT biosynthesis gene tryptophan hydroxylase 1 (TPH1) [13] induces lactation-to-involution switching via 5-HT_7_ receptor-dependent tight junction disruption [18, 19]. Interestingly, whereas 5-HT has a growth inhibitory effect on normal mammary epithelial cells, it stimulates the proliferation of breast cancer cells at sub-micromolar concentrations [20]. 5-HT also stimulates angiogenesis, which involves the proliferation, invasion, and migration of endothelial cells [21–25]. Although the mechanisms responsible for the effects of 5-HT on the malignant phenotype of breast cancer remain unclear, the angiogenesis-promoting effect of 5-HT suggests that it may stimulate cancer cell proliferation and invasion, the required processes of cancer progression [26–28]. In addition, the signaling pathway associated with angiogenic action of 5-HT is known to be mediated by a mechanism similar to that associated with the angiogenic effect of vascular endothelial growth factor (VEGF) (e.g., via the activation of PI3K/Akt signaling) [29], which suggests potential overlap between signaling molecules with respect to cancer proliferation/invasion and angiogenesis.

In the present study, we investigated the role and mode of action of 5-HT in breast cancer progression. In addition, based on our previous results that 6-amino-2,4,5-trimethylpyridin-3-ol analogues inhibit VEGF-induced angiogenesis [30], and that one of the derivatives also blocked angiogenic 5-HT signaling in endothelial cells [31], we examined the therapeutic potential of BJ-1113, one of the aminopyridinol derivatives, which showed greatest antiangiogenic activity, by comparing its abilities to antagonize 5-HT_7_ receptor (SB269970) and to inhibit PI3K/Akt, a common signaling molecule downstream of 5-HT_7_ and VEGF receptors.

## Methods

### Materials

Dulbecco’s modified Eagle’s medium (DMEM) and RPMI1640 were purchased from Hyclone. Cortisone acetate and VEGF were from R&D Systems (Minneapolis, MN, USA). Fetal bovine serum (FBS), penicillin, and streptomycin were obtained from Gibco (Grand Island, NY, USA). Cholera toxin, 2′,2′-dideoxyadenosine (DDA), Forskolin, Pertussis toxin, LY390762, and sodium pyruvate were purchased from Sigma-Aldrich (St. Louis, MO, USA). AZM-475271, Gallein, Cyanopindolol hemifumarate, Cinanserin hydrochloride, and SB-269970 hydrochloride were purchased from Tocris Bioscience (Tocris House, Bristol, UK). Wortmannin was from Biomol (Plymouth Meeting, PA, USA). Antibodies directed against phospho-p85-PI3K (at Tyr488), p85-PI3K, phospho-AKT (at T308), AKT, phospho-mTOR (at Ser2448), mTOR, phospho-ERK, ERK, phospho-p38, p38, VEGF were purchased from Cell Signaling Technology Inc. (Beverly, MA, USA), and β-Actin antibody was purchased from Santa Cruz Biotechnology (Santa Cruz, CA, USA). Tryptophan hydroxylase 1 and MMP-9 antibodies were obtained from Abcam (Cambridge, MA, USA). Antibodies against 5-HT_1D_ and 5-HT_7_ receptors were purchased from Novus Biologicals (Littleton, CO, USA). BJ-1113 was synthesized as described previously [30]; its chemical structure is shown in Fig. 5a. Cisplatin purchased from Shandong Boyuan Pharmaceutical Co. Ltd. (Shandong, China) was dissolved in dimethylsulfoxide (DMSO) to make stock solution and diluted 100 × with 1× Phosphate Buffered Saline (PBS) for in vivo experiments.

### Cell lines and 5-HT treatment

The MCF-10A human breast cell and HCC-1395, T47D, and Hs578T human breast cancer cell lines were purchased from the Korean Cell Line Bank, Seoul. MCF-7 and MDA-MB-231 human breast cancer cells were obtained from the American Type Culture Collection (Manassas, VA, USA). Hs578T, MCF-7 and MDA-MB-231 were grown in DMEM supplemented with 10% FBS and 1% penicillin/streptomycin, whereas T47D, HCC-1395 and MCF-10A were maintained in RPMI1640 supplemented with 10% FBS and 1% penicillin/streptomycin. To measure tumor growth by bioluminescent imaging, MDA-MB-231 cells were retrovirally transduced to express both effluc and Thy1.1. Thy1.1-positive cells were sorted using CD90.1 MicroBeads (Miltenyi Biotec, Auburn, CA, USA). Established stable cells expressing effluc and Thy1.1 are herein referred to as MDA-MB-231/effluc cells and were used to generate tumor-bearing mice.

### Proliferation assay

Cell proliferation was determined using CellTiter 96 Aqueous One Solution (Promega Corporation, WI, USA) according to the manufacturer’s instructions. Cells were seeded in 96-well plates at a density of 2.5 × 10^4^ cells/cm^2^, and on the following day were serum starved overnight in media containing 0.2% FBS. They were then pre-treated with indicated concentration of drugs for 1 h prior to being treated or not with 5-HT or serum. After 72-h of incubation, CellTiter 96 Aqueous One Solution was added and cells were incubated for a further 2 h. Color intensities were measured using microplate reader (Versamax, Molecular Devices, Inc., USA) at 490 nm.

### siRNA transfection

MDA-MB-231 cells plated in 96-well plates (proliferation assay) or 6-well plates (transfection efficiency measurement) were transfected with two different sequences of siRNAs targeting TPH1, 5-HT_1D_, or 5-HT_7_ (Bioneer Corporation, CA, USA) using Dharmacon reagent (Thermo Scientific, MA, USA) when 50% confluent. They were then incubated with transfection mixture consisting of 100 nM siRNA and Dharmafect 1 for 24 h and then with regular media for another 6 h. Cells transfected with TPH1 were used for cell proliferation assays or for VEGF secretion measurements.

### Polymerase chain reaction (PCR)

Total RNA was extracted using Trizol reagent (Invitrogen Corporation, CA, USA) according to the manufacturer’s protocol. cDNA was synthesized using the Goscript reverse transcription system (Promega Corporation, WI, USA). Quantitative mRNA analysis was performed using a QuantiTect SYBR Green PCR kit (Qiagen, CA, USA). The primer sequences used were; 5-HTR1D (sense 5′-TACTGGGCAATCACAGATGC-3′ and antisense 5′-TGGAGATGCAGATGGAGATG-3′) and 5-HTR7 (sense 5′-ACTCTACCGCAGTGGCATTT-3′ and antisense 5′-TGTGTTTGGCAGCACTCTTC-3′). Primers for GAPDH were supplied by Qiagen, CA, USA. RT-PCR was performed in 0.2-mL tubes containing 10× Taq buffer, dNTP, Taq DNA polymerase (Takara, Japan), and 25 pmole of TPH1 primer (forward 5′-ATGATTGAAGACAATAAGGAG-3′ reverse 5′-AAGTTTTTGAGATACTCTCTG-3′) or GAPDH primer (forward 5′-GGTGAAGGTCGGAGTCAACG-3′ and reverse 5′-CAAAGTTGTCATGGATGACC-3′). Amplified PCR products were separated in 1.5% agarose gels, visualized using a gel imaging system (UVP, Cambridge, UK), and quantitated by measuring band densities.

### Determination of VEGF and 5-HT levels

To determine VEGF and 5-HT levels in cells and chick chorioallantoic membrane (CAM) tumor tissues, ELISA kits specific to VEGF (R&D Systems Inc., MN, USA) or 5-HT (IBL America, MN, USA) were used. Cells seeded in 24-well plates at 1 × 10^5^ cells/cm^2^ were serum-starved overnight and treated with BJ-1113 or vehicle in the presence of Hank’s balanced salt solution for 24 h. The cell culture supernatants were collected and saved at −80 °C for 5-HT and VEGF content determination. To determine VEGF and 5-HT contents in CAM tumors, tissues were homogenized in PBS and centrifuged at 900 g for 10 min at 4 °C. 5-HT concentrations in cell culture supernatants and CAM tumor tissues were determined according to the manufacturer’s instructions.

### Protein extraction and western blotting

Whole cell lysates were prepared using radioimmunoprecipitation assay (RIPA) buffer containing 1× protease and phosphatase inhibitor cocktail (Thermoscientific, CA, USA) RIPA buffer was composed of 150 mM Sodium chloride, 1% Triton X-100, 0.5% Sodium deoxycholate, 0.1% SDS and 50 mM Tris adjusted to pH 8.0. After lysing cells with RIPA buffer, they were centrifuged at 16200 g for 15 min and supernatants containing soluble proteins were collected. Protein contents were measured using BCA protein assay reagent (Pierce, Rockford, IL, USA). Equal amounts of total proteins were then separated by SDS-PAGE and transferred onto Hybond ECL nitrocellulose membranes (Amersham Life Science, Buckinghamshire, UK) at 200 mA for an hour. Membranes were blocked in 5% skim milk in Tris-buffered saline (TBS)-Tween 20 (TBS-T) at room temperature for 1 h, incubated in skim milk-TBS overnight at 4 °C, washed three times with TBS-T, and incubated with horseradish peroxidase-conjugated secondary antibody in skim milk-TBS for 1 h at room temperature. Immunoreactive proteins were visualized using an ECL kit (Pierce, Rockford, IL, USA) and digitally processed using LAS-4000 mini (Fuji, Japan). Membranes were stripped and reprobed with an actin antibody as a loading control. Densitometric analysis of blots was performed with Multi Gauge Ver 3.2 imaging software in a Fuji Image Station.

### Invasion assay

Invasion assays were performed as described by Banskota et al., 2015 [32] with slight modification. Briefly, the inner and outer surfaces of Transwell inserts (BD Falcon, Franklin Lakes, USA) were coated with matrigel and collagen, respectively. Serum or 5-HT was added to the lower Transwell chamber except the control group. Cell suspension (100 μl) with or without the indicated inhibitors at indicated concentrations were added to upper Transwell chambers, and 18 h later, cells were fixed and stained. Numbers of cells invading per field were captured at 200× magnification using a digital camera fitted to the microscope.

### cAMP Measurements

MDA-MB-231 cells (1 × 10^5^ cells/cm^2^ in 24 well plate) pretreated with BJ-1113 in DMEM High Glucose medium containing 1% FBS and then stimulated with forskolin, 5-HT, cholera toxin, or pertusis toxin. After washing cells three times with cold PBS, lysis buffer was added to each well, and cells were lysed by freezing (−80 °C for 1 day) and thawing in ice with gentle mixing. Cells were then centrifuged at 600 × g for 10 min at 4 °C to remove cellular debris. Supernatants were assayed for cAMP using a cAMP ELISA kit (R & D Systems Inc., MN, USA).

### CAM assay

Fertilized eggs were purchased from Siprigol Poultry Farm (Gyeongsan, Korea) and the CAM was prepared as described previously [33]. For tumor angiogenesis experiments, MDA-MB-231 human breast cancer cells (1.5 × 10^6^ cells/CAM) were inoculated onto the CAM with or without the indicated concentration of drugs [34, 35]. Numbers of vessel branch points contained in a tumor region were counted by two observers in a double-blind manner. Tumor tissues detached from the membrane were weighed and then preserved at −80 °C for 5-HT ELISA and western blotting.

Chick embryo experiments were performed in accordance with the institutional guidelines of the Institute of Laboratory Animal Resources (1996) and Yeungnam University for the care and use of laboratory animals (2009).

### Anti-tumor activity measurements in mouse tumor models

Six-week-old BALB/c nude mice were purchased from Orient Co. Ltd., Korea and housed under pathogen-free conditions under a 12-h light/dark cycle with access to food pellets and tap water *ad libitum*. Mice were injected subcutaneously with 1 × 10^7^ MDA-MB-231-effluc cells in 200 μl of DMEM/matrigel (1:1) into a flank. Tumors were measured every two days and volumes were calculated using the formula:

$$ \mathrm{V}\ \left({\mathrm{mm}}^3\right) = \mathrm{Length}/2 \times {\left(\mathrm{Width}\right)}^2 $$

When tumors reached 200 mm^3^, mice were divided into different groups and injected intraperitoneally with drugs once daily. Mice were monitored daily for physical condition (body weight changes and rustling behavior). After 19 days of treatment, mice were sacrificed in a CO_2_ gas-filled chamber, and the excised tumors were measured and weighed.

For in vivo bioluminescent imaging (BLI), mice were administered d-luciferin i.p. injection. After 10 min of D-luciferin injection, BLI was performed using the IVIS Lumina III imaging system (PerkinElmer, MA, USA). Grayscale photographic images and bioluminescent color images were superimposed using LIVINGIMAGE (version 2.12, PerkinElmer) and IGOR Image Analysis FX software (WaveMetrics, Lake Oswego, OR, USA). BLI signals are expressed as photons per cm^2^ per second per steradian (P/cm^2^/s/sr). All mice were anesthetized using 1–2% isoflurane gas during imaging.

Mouse experiments were performed according to the institutional guidelines of the Institute of Laboratory Animal Resources (1996) and of Yeungnam University for the care and use of laboratory animals (2009). The guidelines also contain a protocol for early euthanasia/humane endpoints for mice that become severely ill prior to the experimental endpoint. If a mouse lost 20% of its body weight due to drug treatment or gained 20% of body weight due to tumor growth, it was euthanized early. The study protocol was reviewed and approved beforehand by the Institutional Animal Care and Use Committee of Yeungnam University.

### Statistical analysis

Data are presented as means ± SEMs. Statistical significance was determined using the Student’s *t*-test or one-way Analysis of variance (ANOVA) followed by the Newman-Keuls comparison method in GraphPad Prism 5.0 software (San Diego, CA, USA). *P* values of less than 0.05 were considered statistically significant.

## Results

### The autocrine effect of 5-HT on MDA-MB-231 human breast cancer cell proliferation was mediated through 5-HT_7_ receptor

**Fig. 1:**
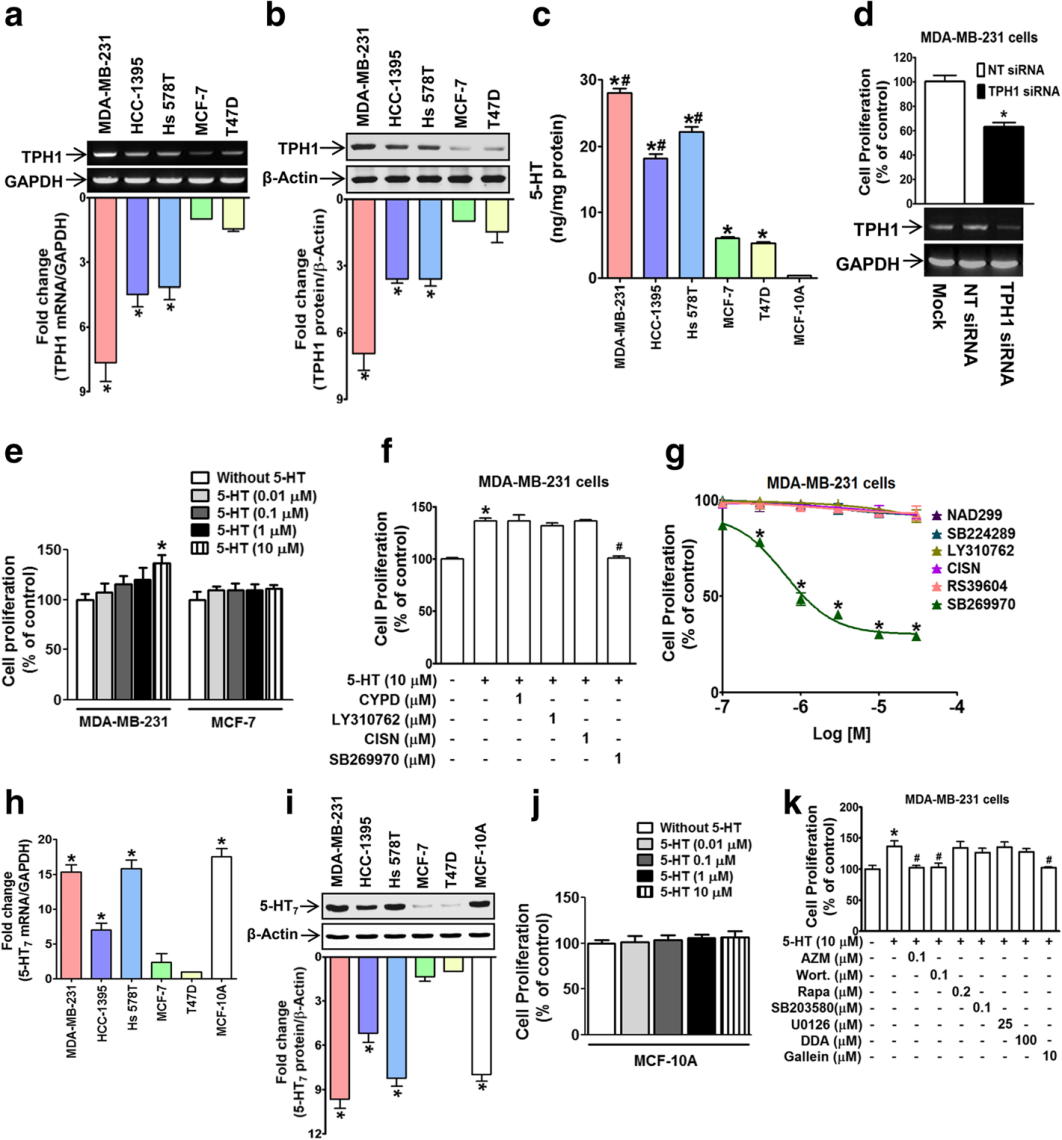
Autocrine action of 5-HT in MDA-MB-231 human breast cancer cell proliferation and its mediation through 5-HT_7_ receptor. **a**, **b** The mRNA (**a**) and protein (**b**) expression levels of TPH1 expression in TNBCs (MDA-MB-231, HCC-1395, and Hs578T) and hormone-responsive breast cancer cells (MCF-7 and T47D). The bar graph represents relative densities of TPH1 (TPH1 mRNA/GAPDH or TPH1 protein/β-Actin) normalized versus those in MCF-7 cells. Results are the means of three independent experiments. *P < 0.05 vs. MCF-7 cells. **c** 5-HT secretion in TNBCs, hormone-responsive breast cancer cells and MCF-10A human normal breast cells was measured by ELISA. *P < 0.05 vs. MCF-10A cells, ^#^ *P* < 0.05 vs. T47D cells. **d** Transfection of MDA-MB-231 cells with TPH1 siRNA suppressed proliferation. The bands underneath the bar graph represent TPH1 mRNA expressions detected by PCR. *P < 0.05 vs. non-target (NT) siRNA-transfected controls. **e** Comparison of cell proliferation induced by 5-HT in MDA-MB-231 and MCF-7 human breast cancer cells. *P < 0.05 vs. vehicle-treated controls. **f** 5-HT-treated MDA-MB-231 proliferation was examined in the presence of cyanopindolol (CYPD), LY310762, cinanserin (CISN), or SB269970. *P < 0.05 vs. vehicle-treated controls. ^#^ *P* < 0.05 vs. 5-HT-treated cells. **g** MDA-MB-231 cell proliferation in the presence of serum was inhibited by 5-HT_7_ antagonist. *P < 0.05 vs. vehicle-treated controls. **h**, **i** The mRNA expression determined by real time PCR (**h**) and protein expression (**i**) of 5-HT_7_ receptors in TNBCs (MDA-MB-231, HCC-1395, and Hs 578 T), hormone-responsive breast cancer cells (MCF-7 and T47D), and MCF-10A normal breast cells. *P < 0.05 vs. T47D cells. **j** Effect of 5-HT on MCF-10A cell proliferation. **k** Effect of 5-HT receptor-downstream signaling inhibitors on 5-HT-stimulated cell proliferation. *P < 0.05 vs. vehicle-treated controls. ^#^ *P* < 0.05 vs. 5-HT-treated cells

To determine whether 5-HT exerts a mitogenic signal to TNBCs in an autocrine manner, we first measured the expression levels of TPH1, the 5-HT synthesizing enzyme, in cells. TNBC cells (MDA-MB-231, HCC-1395, Hs578T) expressed TPH1 higher at the mRNA (Fig. 1a) and protein levels (Fig. 1b) than hormone-responsive cells (MCF-7 and T47D). Likewise, 5-HT secretion by TNBCs, which was measured in Hank's balanced salt solution without serum, was much higher than that secreted by hormone-responsive cells or normal breast cell line (MCF-10A) (Fig. 1c). Knock-down of TPH1 expression using siRNA significantly reduced the proliferation of MDA-MB-231 cells (Fig. 1d). Furthermore, exogenous 5-HT application (in the absence of serum) stimulated MDA-MB-231 cell proliferation, but this mitogenic action was not observed in MCF-7 cells (Fig. 1e), possibly due to differences in 5-HT signaling pathways. To identify the 5-HT receptors mediating its mitogenic effect, the proliferation of MDA-MB-231 cells was examined in the presence of inhibitors of different 5-HT receptors. The mitogenic effect of 5-HT was blocked by SB269970 (a 5-HT_7_ antagonist), but not by cyanopindolol (a 5-HT_1A_ antagonist), LY310762 (a 5-HT_1D_ antagonist), or cinanserin (a 5-HT_2A/2C_ antagonist) (Fig. 1f). In addition, MDA-MB-231 proliferation in the presence of serum was blocked by SB269970, but not by NAD299 (a 5-HT_1A_ antagonist), SB224289 (a 5-HT_1B_ antagonist), LY310762, cinanserin, or RS39604 (a 5-HT_4_ antagonist) (Fig. 1g), suggesting 5-HT_7_ receptor plays a major role in the mitogenic effect of autocrine 5-HT. To further explain the cell-specific mitogenic action of 5-HT, we examined 5-HT_7_ receptor expression in multiple breast cancer cell lines. The mRNA (Fig. 1h) and protein (Fig. 1i) expression levels of 5-HT_7_ receptor in TNBCs (including MDA-MB-231 cells) were much higher than in MCF-7 and T47D cells. In addition, in MCF-10A normal breast cells which express high level of 5-HT_7_ receptor (Fig. 1h and i), 5-HT did not stimulate the cell proliferation (Fig. 1j). These results indicate that the mitogenic effect of 5-HT is TNBC cell line specific. We also examined 5-HT_7_ downstream signaling involved in 5-HT-induced proliferation in MDA-MB-231 cells. 5-HT-induced proliferation was suppressed by inhibitors of Src (AZM-475271), PI3K (wortmannin), and gallein (a Gβγ inhibitor), but not by inhibitors of adenylyl cyclase (DDA), mTOR (rapamycin), p38 (SB203580), or MAPKK (U0126) (Fig. 1k).

### 5-HT stimulated MDA-MB-231 cell invasion via a 5-HT_7_ receptor-dependent signaling pathway

**Fig. 2:**
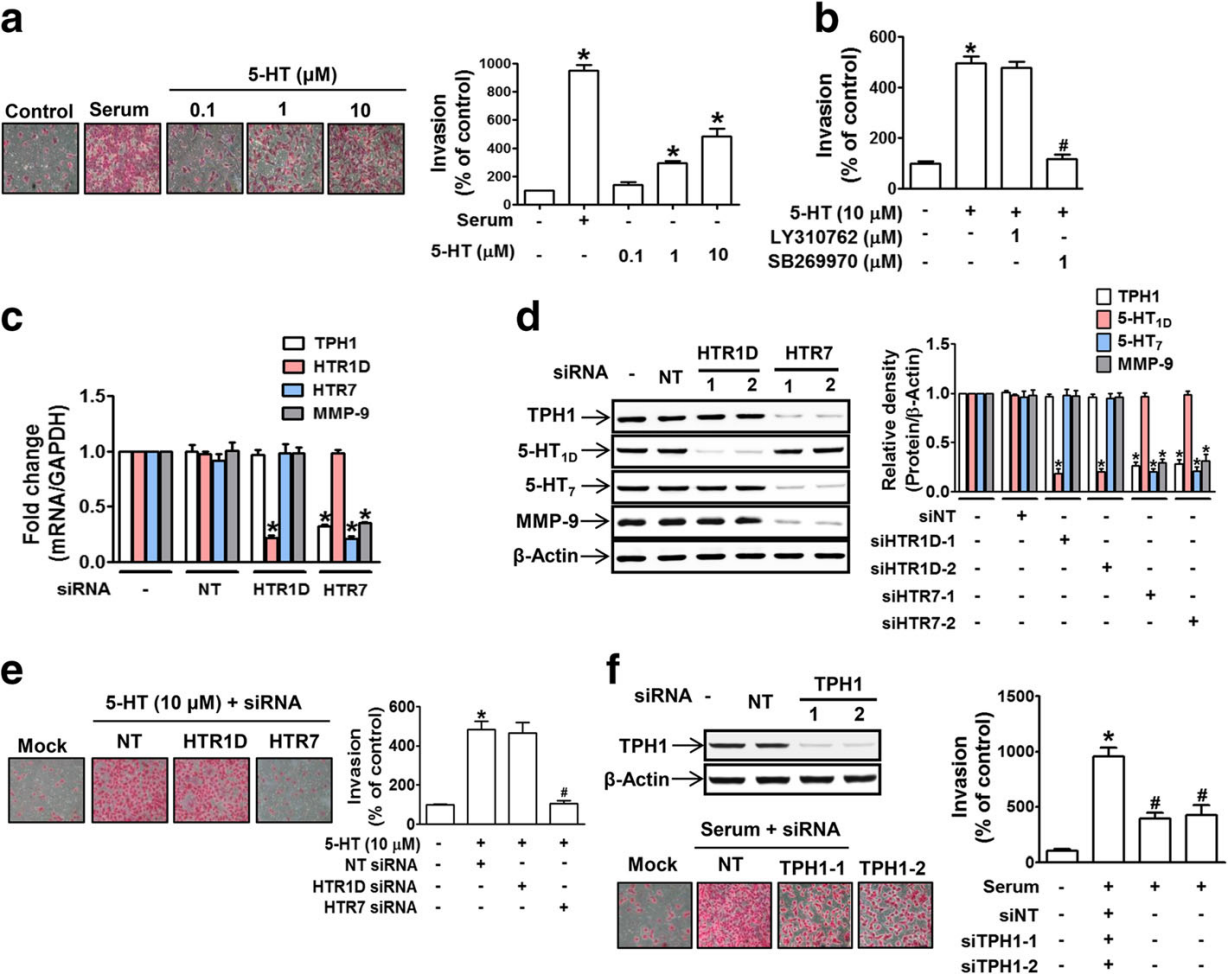
5-HT stimulated MDA-MB-231 cell invasion through 5-HT_7_ receptors. **a** MDA-MB-231 cells seeded in a Matrigel-coated transwell inserts were serum-starved and then treated with 5-HT for 18 h. Serum was used as a positive control to induce invasion. Invading cells were counted in five different fields per well, and results were averaged. The bar graphs represent the relative numbers of invading cells expressed as the means ± SEMs of three independent experiments. *P < 0.05 vs. vehicle-treated controls. **b** Cells were pretreated with LY310762 or SB269970 for 1 h prior to 5-HT (10 μM) treatment. *P < 0.05 vs. vehicle-treated controls. ^#^ *P* < 0.05 vs. 5-HT-treated cells. **c**-**e** MDA-MB-231 cells were transfected with two different sequences of siRNAs targeting either NT, 5-HT_1D_, or 5-HT_7_ receptor. The bar graphs represent mRNA and protein expression levels of TPH1, 5-HT_1D_, 5-HT_7_, and MMP-9, as determined by real time PCR (**c**) and western blot (**d)** *P < 0.05 vs. NT siRNA-transfected controls. **e** 5-HT-induced invasion ability was measured by invasion assay. *P < 0.05 vs. vehicle-treated controls. ^#^ *P* < 0.05 vs. cells treated with NT siRNA and 5-HT. **f** Serum-induced invasion was examined in MDA-MB-231 cells transfected with NT or two different sequences of TPH1 siRNA. *P < 0.05 vs. vehicle-treated controls. ^#^ *P* < 0.05 vs. NT siRNA-transfected cells

We examined whether 5-HT promotes tumor progression by inducing breast cancer cell invasion. 5-HT stimulated MDA-MB-231 cell invasion in a concentration-dependent manner at concentrations as low as 1 μM (Fig. 2a). To determine whether 5-HT_7_ receptor mediates 5-HT-induced invasion, we examined whether SB269970 (a 5-HT_7_ receptor antagonist) prevents cancer cell invasion. An inhibitor of 5-HT_1D_ (LY310762) was included as a negative control because it has been reported 5-HT_1D_ is highly expressed in MDA-MB-231 cells [20]. 5-HT-enhanced MDA-MB-231 cell invasion was found to be blocked by SB269970 but not by LY310762 (Fig. 2b), indicating 5-HT_7_ receptor plays a key role in 5-HT-induced invasion. To confirm the role of 5-HT_7_ receptor in cell invasion, 5-HT_7_ receptor expression was knocked-down using siRNA_._ Silencing of 5-HT_7_ (but not of 5-HT_1D_) receptor (Fig. 2c and d) abrogated 5-HT-induced MDA-MB-231 cell invasion (Fig. 2e), and this was accompanied by a decrease in the expression of matrix metalloproteinase-9 (MMP-9), a positive regulator of cancer cell invasion (Fig. 2c and d). Of note, 5-HT_7_ knock-down also reduced the expression of TPH1 (Fig. 2c and d). To estimate the potential contribution of autocrine 5-HT to cell invasion, we knocked down TPH1 expression using TPH1 siRNA, and it was found that this inhibited the invasion of MDA-MB-231 cells in the presence of serum (Fig. 2f), suggesting that the important autocrine function of 5-HT during MDA-MB-231 cancer cell invasion.

**Fig. 3:**
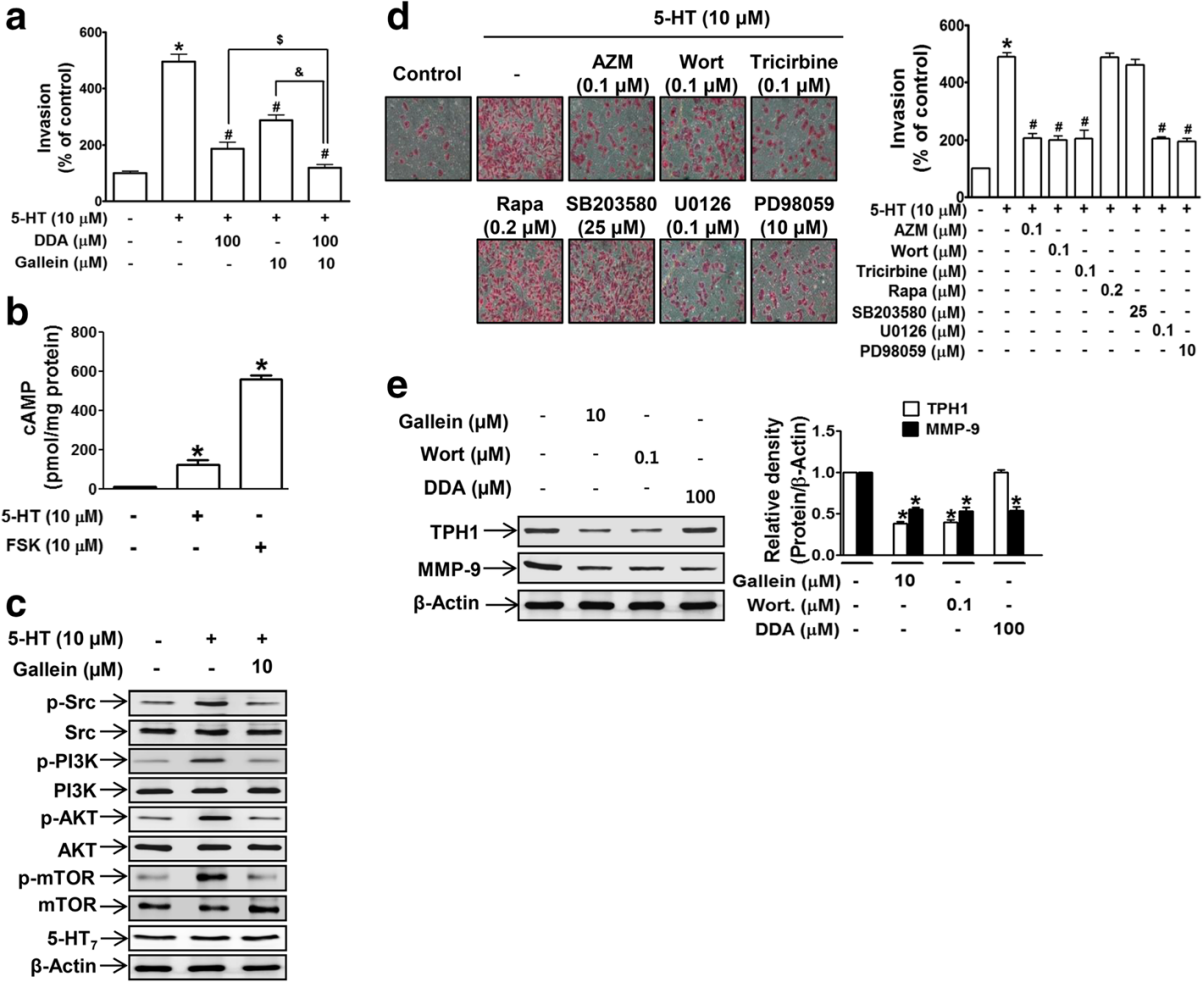
5-HT-induced MDA-MB-231 cell invasion was linked to 5-HT_7_ receptor-dependent Gαs and Gβγ signaling. **a** The invasivenesses of MDA-MB-231 cells were determined after cells were pretreated with gallein or DDA alone, or a combination of both, for 1 h prior to 5-HT treatment. *P < 0.05 vs. vehicle-treated controls. ^#^ *P* < 0.05 vs. 5-HT-treated cells. ^$^ *P* < 0.05 vs. 5-HT- and DDA-treated cells. &*P* < 0.05 vs. 5-HT- and gallein-treated cells. **b** Cells were treated with 5-HT or forskolin and cAMP levels were measured. *P < 0.05 vs. vehicle-treated controls. **c** Protein extracts from MDA-MB-231 cells treated with 5-HT alone or 5-HT plus gallein were analyzed for the activation of Gβγ-downstream signaling by western blotting. **d** 5-HT-induced MDA-MB-231 cell invasion was examined in the presence of various inhibitors, that is, AZM-475271 (AZM), wortmannin (Wort), tricirbine, rapamycin (Rapa), SB203580, U0126, and PD98059, at the indicated concentrations. *P < 0.05 vs. vehicle-treated controls. ^#^ *P* < 0.05 vs. 5-HT-treated cells. **e** Cells were treated with gallein, wortmannin, or DDA, and basal levels of TPH1 and MMP-9 were analyzed by western blotting. *P < 0.05 vs. vehicle-treated controls

We next investigated signaling molecules downstream of 5-HT_7_ receptor involved in cancer cell invasion. First, to identify G protein component(s) responsible for the effect of 5-HT, invasion assays were performed in the presence of G protein component-specific inhibitors, that is, DDA (a Gαs-linked adenylate cyclase inhibitor) or gallein (a Gβγ inhibitor). 5-HT-stimulated cell invasion was significantly suppressed by DDA and by gallein (Fig. 3a). In addition, co-treatment with DDA and gallein completely stopped 5-HT-induced invasion (Fig. 3a), suggesting that Gαs and Gβγ mediate 5-HT-induced cancer cell invasion in concert. As was expected from the involvement of Gαs in 5-HT-induced invasion, intracellular cAMP levels were increased after treating MDA-MB-231 cells with 5-HT, but notably, this increase in cAMP was much smaller than that induced by forskolin (the activator of adenylyl cyclase) (Fig. 3b), suggesting Gαs of 5-HT_7_ receptor may not be fully functional at the basal level. Downstream signaling molecules of Gβγ, that is, Src, PI3K (p85), Akt, and mTOR, were found to be activated (phosphorylated) in 5-HT-treated cells, and these activations were inhibited by gallein (Fig. 3c), which is consistent with the involvement of Gβγ in 5-HT-induced cell invasion. To confirm the roles of these signaling pathways in 5-HT-induced cancer cell invasion, invasion assays were performed using MDA-MB-231 cells in the presence of inhibitors of different signaling pathways. The 5-HT-induced invasion of MDA-MB-231 cancer cells was blocked by inhibitors of Src (AZM-475271), PI3K (wortmannin), Akt (tricirbine), MAPKK (U0126), or ERK (PD98059) (Fig. 3d), but interestingly, not by inhibitors of mTOR (rapamycin) or p38 (SB203580) (Fig. 3d), indicating that not all activated Gβγ signaling molecules were involved in the 5-HT-induced invasion. To determine the role of signaling molecules in the altered expressions of downstream genes involved in 5-HT-induced invasion, we examined the expression levels of MMP-9 and TPH1 in the presence of inhibitors of signaling pathways including, Gαs (DDA), Gβγ (gallein), and PI3K (wortmannin). Basal TPH1 expression was suppressed by gallein and wortmannin, but not by DDA (Fig. 3e), whereas all three inhibitors suppressed MMP-9 expression (Fig. 3e), suggesting that the signaling pathway linked to MMP-9 expression was different from that required for TPH1 expression.

### 5-HT crosstalk with VEGF and the induction of VEGF expression by 5-HT: Suppression by BJ-1113

**Fig. 4:**
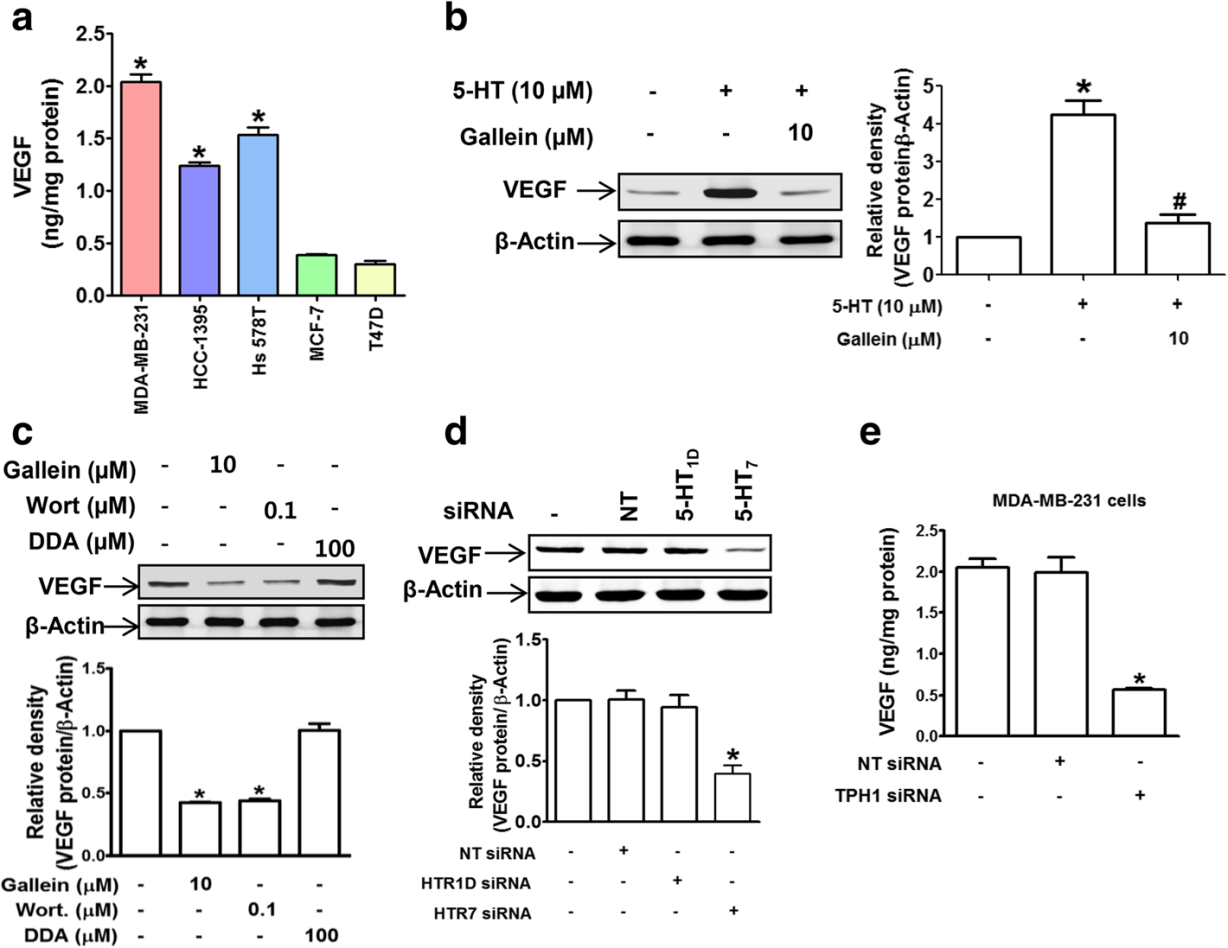
5-HT crosstalks with VEGF by inducing its expression. **a** VEGF secretions by TNBCs and hormone-responsive breast cancer cells were measured by ELISA. *P < 0.05 vs. T47D cells. **b** Cells were treated as described in the legend of Fig. 3c and VEGF protein levels were determined by western blotting. *P < 0.05 vs. vehicle-treated controls. ^#^ *P* < 0.05 vs. 5-HT-treated cells. **c** Basal levels of VEGF protein in MDA-MB-231 cells treated with gallein, wortmannin, or DDA. The bar graphs represent the means ± SEMs of three independent experiments. *P < 0.05 vs. vehicle-treated controls. **d** Knockdown of 5-HT_7_ suppressed VEGF protein expression. *P < 0.05 vs. vehicle- or NT siRNA-treated controls. **e** Knock-down of TPH1 inhibited VEGF secretion by MDA-MB-231 cells. *P < 0.05 vs. vehicle- or NT siRNA-transfected controls

Previous studies have suggested potential overlap between the 5-HT and VEGF signaling pathways [29], both of which apparently promote cancer cell growth and survival. To determine whether there indeed is overlap between these signaling pathways, we examined whether VEGF expression is altered by 5-HT or inhibitors of signaling pathways involved in the effects of 5-HT. VEGF secretion from TNBCs in the absence of serum was much higher than from hormone-responsive cells (Fig. 4a), and its expression was increased in the presence of 5-HT (Fig. 4b). Furthermore, 5-HT-induced VEGF expression was suppressed in the presence of inhibitors of Gβγ and PI3K (Fig. 4c). In addition, knock-down of 5-HT_7_ or TPH1 led to significant decreases in VEGF protein expression (Fig. 4d) and VEGF concentrations in culture media (Fig. 4e), respectively. Together, these results indicate overlapping between the 5-HT and VEGF signaling pathways and suggest potential crosstalk between the two.

**Fig. 5:**
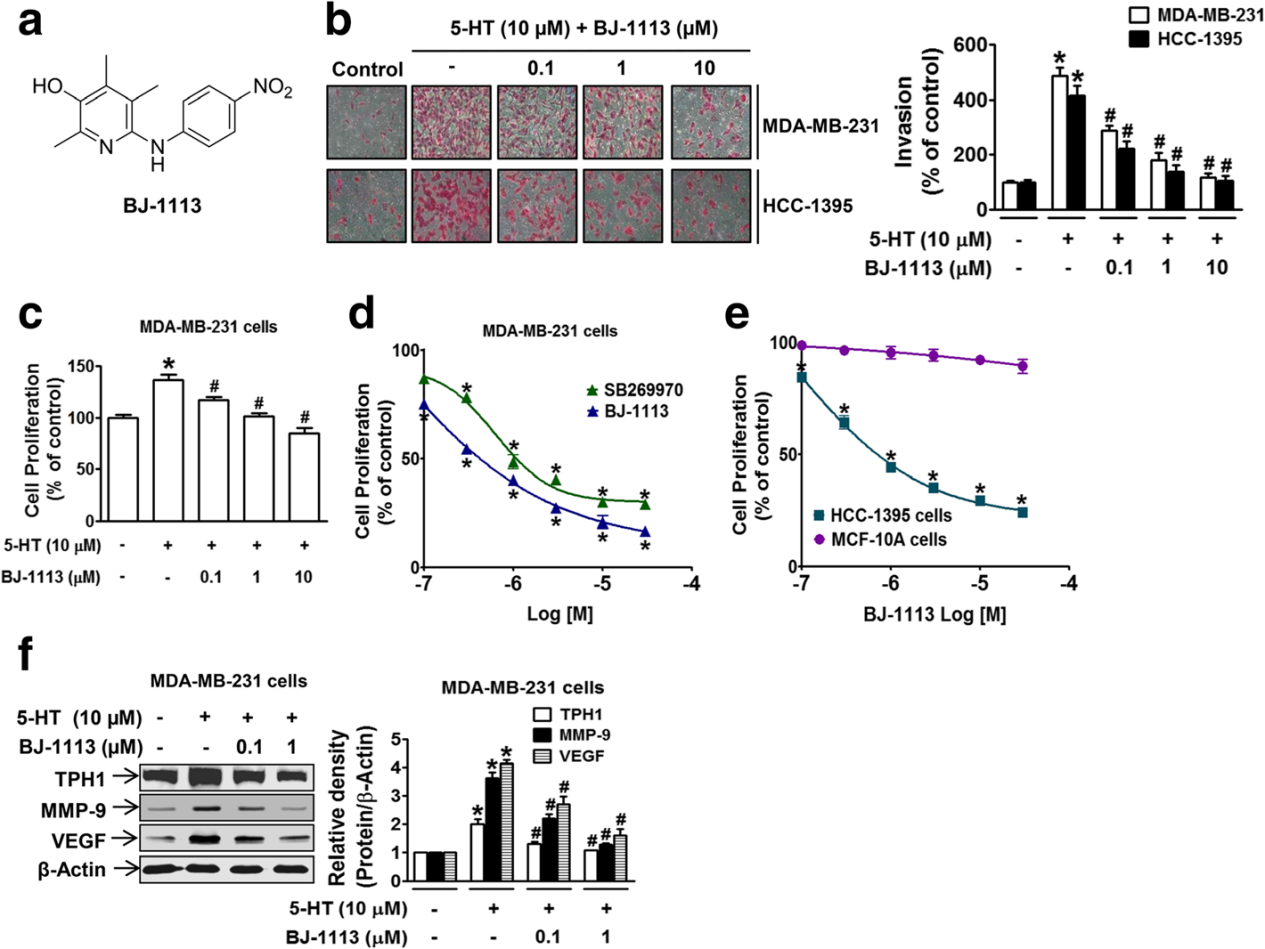
Inhibitory effects of BJ-1113 on 5-HT-induced invasion and proliferation of MDA-MB-231 cells. **a** Chemical structure of BJ-1113. **b** Treatment of MDA-MB-231 cells and HCC-1395 cells with BJ-1113 concentration-dependently suppressed 5-HT-induced invasion. **c** Measurements of MDA-MB-231 proliferation in the absence or presence of BJ-1113. **d** Serum-induced MDA-MB-231 cell proliferation was significantly inhibited by BJ-1113, and this was greater than the inhibition observed for SB269970. e BJ-1113 suppressed serum-induced proliferation in HCC-1395, TNBC cells but not in MCF-10A normal breast cells. **f** Western blot analyses of 5-HT-induced TPH1, MMP-9 and VEGF expressions in BJ-1113-treated MDA-MB-231 cells. *P < 0.05 vs. vehicle-treated controls. ^#^ *P* < 0.05 vs. 5-HT-treated cells

To examine the possibility of crosstalk, we next examined whether BJ-1113 exerts an inhibitory effect on 5-HT-induced TNBC progression. BJ-1113 concentration-dependently inhibited 5-HT-induced MDA-MB-231 cell invasion (Fig. 5b) and proliferation (Fig. 5c), and serum-induced TNBC proliferation was significantly inhibited by BJ-1113 more so than by SB269970 (Fig. 5d). The inhibitory effects of BJ-1113 on invasion (Fig. 5b) and proliferation (Fig. 5e) were similarly observed in the other TNBC cell line, HCC-1395. Similar to 5-HT which had no mitogenic effect on MCF-10A cells, BJ-1113 did not inhibit MCF-10A cell proliferation (Fig. 5e). To elucidate the molecular mechanism involved, the expressions of TPH1, MMP-9, and VEGF were examined in BJ-1113-treated MDA-MB-231 cells. Treatment with BJ-1113 significantly suppressed the 5-HT-enhanced expressions of TPH1, MMP-9, and VEGF (Fig. 5f), and consequently, the secretions of VEGF and 5-HT (Table 1), suggesting that BJ-1113 suppresses cancer progression by inhibiting VEGF expression and the autocrine function of 5-HT.

**Table 1:** Secreted levels of 5-HT and VEGF from MDA-MB-231 cells in culture and their inhibition by BJ-1113

		VEGF (ng/mg protein)	5-HT (ng/mg protein)
Vehicle		1.9 ± 0.2	28.6 ± 0.4
BJ-1113 (μM)	0.1	1.3 ± 0.1	18.0 ± 0.8
	1	1.1 ± 0.2	13.9 ± 0.4

**Fig. 6:**
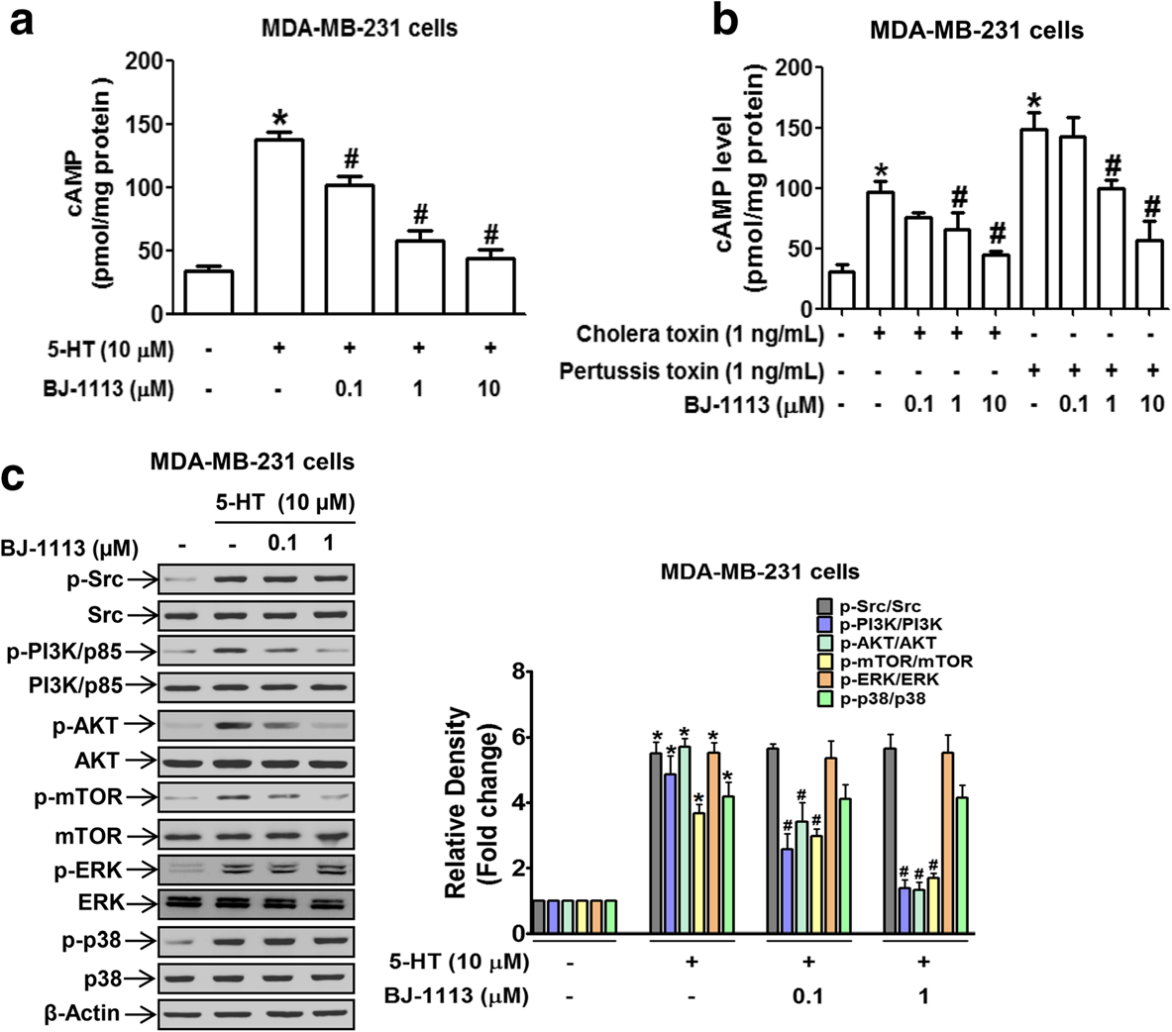
Inhibitory effect of BJ-1113 on Gαs and Gβγ signaling in MDA-MB-231 breast cancer cells. **a**, **b** Inhibitory effects of BJ-1113 on cAMP levels were investigated using a cAMP ELISA kit. In **a**, cAMP level was measured in cells treated with 5-HT in the absence or presence of BJ-1113. *P < 0.05 vs. vehicle-treated controls. ^#^ *P* < 0.05 vs. 5-HT-treated cells. In **b**, BJ-1113 inhibited cholera toxin- and pertusis toxin-induced cAMP in MDA-MB-231 cells. *P < 0.05 vs. vehicle-treated controls. ^#^ *P* < 0.05 vs. cholera toxin- or pertusis toxin-treated cells. **c** Inhibitory effect of BJ-1113 on the activation of Gβγ-downstream signaling in 5-HT-stimulated MDA-MB-231 cells. The results in bar graphs represent the relative densities of each signaling protein of phospho-to-parent and are the means ± SEMs of three independent experiments. *P < 0.05 vs. vehicle-treated controls. ^#^ *P* < 0.05 vs. 5-HT-treated cells

To investigate the molecular mechanism and identify the signaling molecules responsible for the action of BJ-1113, cAMP levels (indicative of Gαs signaling) were measured. In MDA-MB-231 cells, BJ-1113 concentration-dependently suppressed 5-HT-induced cAMP generation (Fig. 6a). We also noted the possibility that Gαs of 5-HT_7_ receptor might not be fully functional at basal level (Fig. 3b). To clarify the involvement of G protein in determining the action of BJ-1113, MDA-MB-231 cells were treated with an activator of Gαs (cholera toxin) or an inhibitor of Gαi (pertussis toxin) and intracellular cAMP levels were measured. As was expected, cholera toxin increased cAMP levels, and pertussis toxin enhanced cAMP levels more so than cholera toxin (Fig. 6b). BJ-1113 consistently suppressed both cholera toxin- and pertussis toxin-induced cAMP increases (Fig. 6b), indicating that action site of BJ-1113 may be adenylyl cyclase rather than G protein. In terms of 5-HT-activated Gβγ signaling, BJ-1113 significantly inhibited the phosphorylations of PI3K, Akt, and mTOR, but not of Src, ERK, and p38 (Fig. 6c).

### Anti-tumor activities of 5-HT_7_ receptor antagonist, its signaling inhibitors, and of BJ-1113 in the MDA-MB-231 xenograft models

**Fig. 7:**
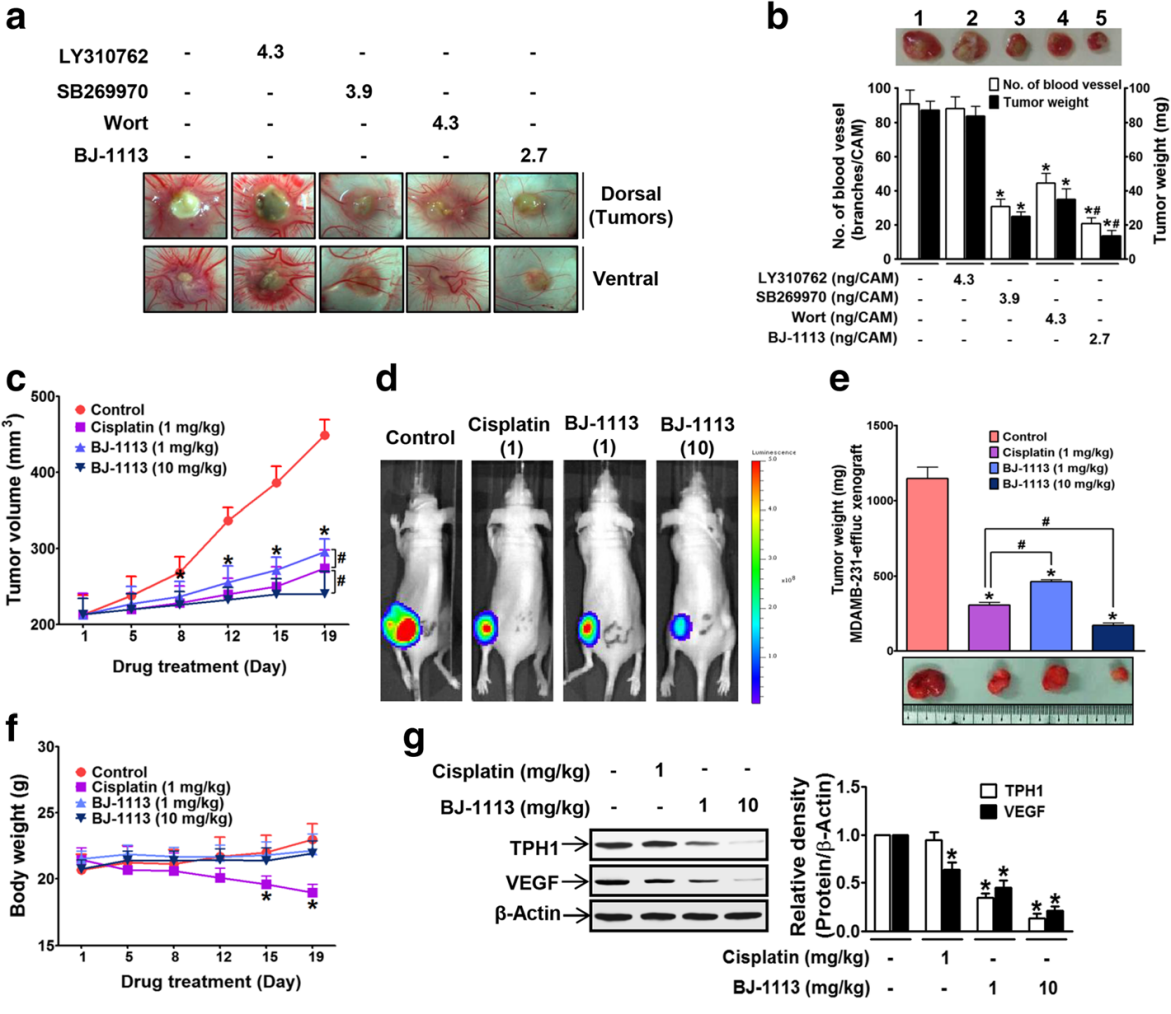
The inhibitory effects of BJ-1113 on xenografted MDA-MB-231 tumor growth on CAM and in nude mice. **a**, **b** MDA-MB-231 human breast cancer cells (2 × 10^6^ cells/CAM) were inoculated on top of CAM; drug were mixed with cell suspension before inoculation. The tumor growth and angiogenesis shown in **a** were quantitated and are expressed as the numbers of new vessel branches formed and tumor weights (**b**). A representative tumor mass isolated from CAM: 1, vehicle control; 2, LY310762-treated group; 3, SB269970-treated group; 4, wortmannin (Wort) treated group; and 5, BJ-1113-treated group. *P < 0.05 vs. PBS-treated controls. ^#^ *P* < 0.05 vs. SB269970 or Wort treated cells. **c**-**e**. BALB/c nude mice bearing MDA-MB-231-effluc cells were administered intraperitoneally with cisplatin or BJ-1113 for 19 consecutive days. In each group, six mice were used. **c** Tumor growth was monitored by measuring tumor size. **d** Bioluminescent imaging of tumors was performed as described in Material and Methods; in addition, **e** tumor tissues were isolated and their weights were recorded. *P < 0.05 vs. vehicle-treated control mice. ^#^ *P* < 0.05 vs. cisplatin-treated mice. **f** Unlike cisplatin, treatment with BJ-1113 for 19 days did not alter body weights. **g** BJ-1113 inhibited TPH1 and VEGF protein expression in MDA-MB-231 effluc tumor. *P < 0.05 vs. vehicle-treated control mice

MDA-MB-231 cancer cell-inoculated chick chorioallantoic membrane (CAM) assay was used to examine the in vivo efficacies of SB269970 (5-HT_7_ receptor inhibitor), wortmannin (an inhibitor of PI3K which is involved in the signaling of 5-HT_7_ and VEGF receptors), and BJ-1113. 5-HT_1D_ receptor antagonist (LY310762) was used as a negative control. MDA-MB-231 cancer cells inoculated onto CAMs developed into tumor masses that exhibited high degrees of new blood vessel formation, and this vessel formation was significantly suppressed by SB269970, wortmannin, and BJ-1113 (Fig. 7a and b). As was expected based on the anti-invasive and anti-proliferative effects of BJ-1113 (Fig. 5), BJ-1113 had the strongest inhibitor effect followed by SB269970 and wortmannin (Fig. 7b). To confirm the anticancer activity of BJ-1113, we also examined its antitumor effects in mice xenografted with MDA-MB-231-effluc cells and compared these with those of cisplatin. Because preliminary results showed cisplatin at 3 mg/kg was highly toxic, we administered cisplatin at 1 mg/kg. BJ-1113 dose-dependently suppressed tumor growth (Fig. 7c), although at 1 mg/kg, its inhibitory effect was slightly less than that of cisplatin. In vivo optical imaging also revealed that BLI signals emitted by tumors were much lower in cisplatin- or BJ-1113-treated mice than in vehicle-treated mice (Fig. 7d). Of note, in vivo BLI imaging revealed greatest inhibition of tumor growth in mice treated with 10 mg/kg BJ-1113. On day 19 after first drug treatment, the effect of BJ-1113 at 10 mg/kg on tumor growth was dramatic, as 53% decrease in tumor volume and 85% decrease in tumor weight was observed in the BJ-1113-treated group (mean tumor volume 240.20 ± 29.53 mm^3^, tumor weight 171.7 ± 38.7 mg) as compared with vehicle-treated controls (mean tumor volume 448.87 ± 20.30 mm^3^, tumor weight 1146.7 ± 183.05 mg) (Fig. 7c and e). However, the body weights (a marker of toxicity) of BJ-1113-treated mice were similar to those of vehicle-treated controls, whereas cisplatin-treated mice showed progressive body weight loss (Fig. 7f). In addition, compared to vehicle-treated control group, TPH1 and VEGF protein expression was suppressed by BJ-1113 in MDA-MB-231 effluc tumor (Fig. 7g).

## Discussion

The present study shows, for the first time, that autocrine 5-HT stimulates TNBC progression. This conclusion is based on the observations that; 1) TNBCs (MDA-MB-231, HCC-1395, and Hs578T) expressed higher levels of TPH1 and 5-HT_7_ receptor, and secreted more 5-HT than hormone-positive cells (MCF-7 and T47D), 2) silencing of TPH1 or 5-HT_7_ receptor blocked cancer invasion and proliferation.

Invasion and proliferation are mutually incompatible phenotypes and are required for the malignant progression of cancer, during which proliferation after invasion supports the malignant process by maintaining cell numbers. These processes could be achieved if different sub-clones of phenotypes with proliferative, invasive, or both phenotypes co-exist in a single tumor [26–28]. Likewise, the present study shows MDA-MB-231 cells in culture responded to 5-HT stimulation by increasing their invasive and proliferative abilities, which indicates MDA-MB-231 cells in culture possessed both proliferative and invasive phenotypes. In addition, the stimulatory effect of 5-HT on MDA-MB-231 cell invasion (Fig. 2a) was found to be greater than its effect on cell proliferation (Fig. 1e), which suggests that 5-HT acts more strongly during the first stage of the metastatic process, that is by promoting invasion and migration through extracellular matrix, rather than during the later proliferative phase after local invasion or migration to new sites. Of note, we identified the downstream signaling molecules responsible for transmitting signals from 5-HT_7_ receptor that lead to either TNBC cell proliferation or invasion.

**Fig. 8:**
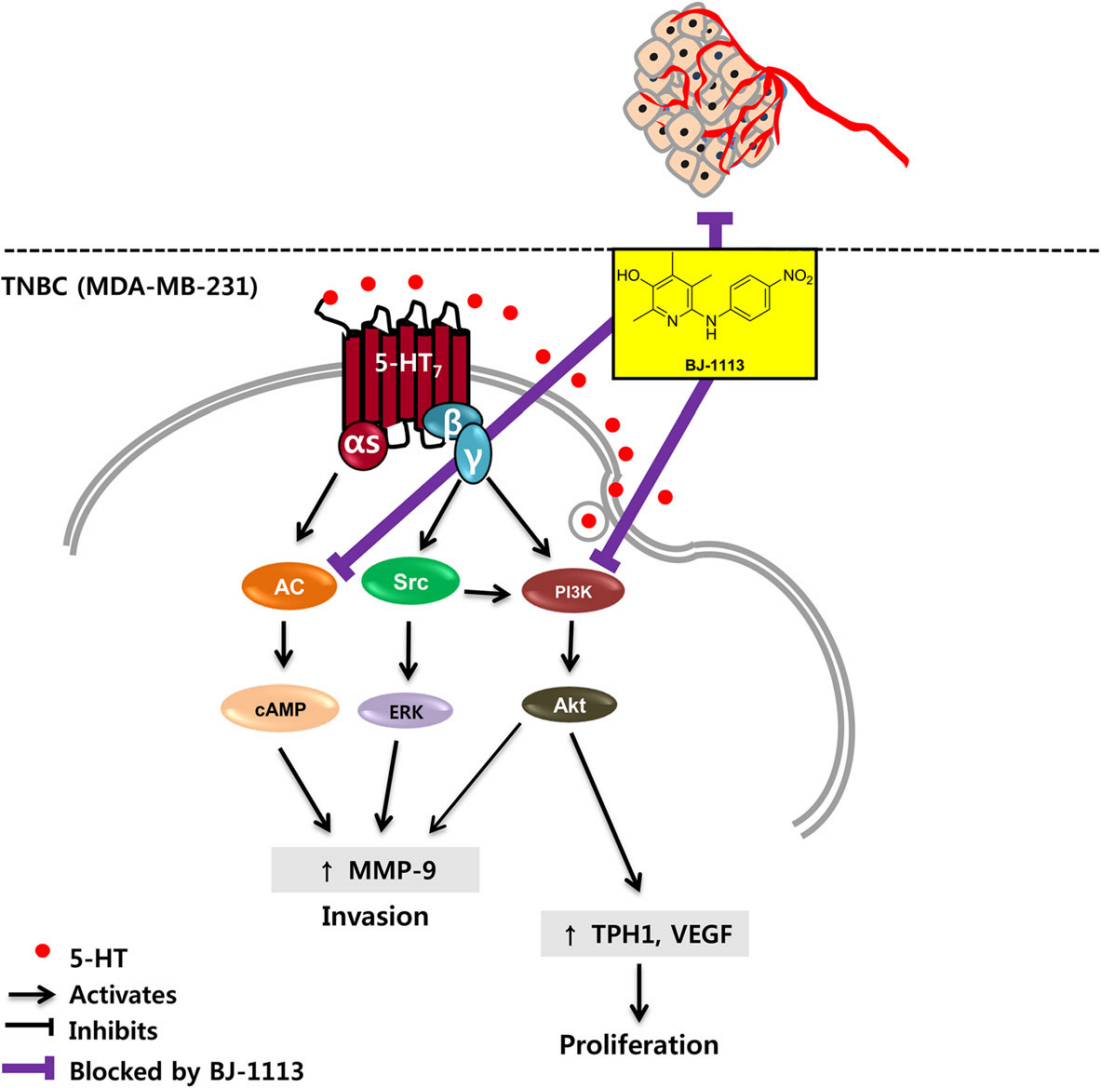
Schematic diagram of 5-HT signaling and of the mode of action of BJ-1113 during breast cancer progression

In a previous study, it was reported MDA-MB-231 cells express 5-HT_1D_ and 5-HT_7_ receptors [20], but in the present study only 5-HT_7_ receptor was found to be associated with their invasion and proliferation. Silencing and treatment with 5-HT_7_ receptor inhibitor suppressed 5-HT-induced breast cancer progression. Furthermore, increased TPH1 expression was maintained by autocrine 5-HT in TNBC cells, which is very different from MCF-10A normal breast cell line. In MCF-10A cells which barely produced 5-HT despite high level expression of 5-HT_7_ receptors, 5-HT had no mitogenic effect on the cells, which is consistent to previous reports [20]. These results suggest that 5-HT signaling via 5-HT_7_ receptor offers a promising therapeutic target for TNBC. Consistent with a previous report that cellular signaling in an invasive subclone of cancer cells is dependent on mitogen-activated protein kinase (MAPK) [26], we observed 5-HT-activated ERK was involved in invasion, but not in proliferation. In contrast, PI3K/Akt, the other downstream signal of 5-HT_7_ receptor-linked Gβγ activation, was involved in the invasion and proliferation of MDA-MB-231 cells. Although PI3K/Akt is also a downstream signaling molecule of growth factor receptors, PI3K inhibitors that block the signal overlap between 5-HT and growth factors may not be enough to suppress TNBC progression fully. However, complete blockade of 5-HT-induced MDA-MB-231 cell invasion occurred when the activities of both Gαs and Gβγ were inhibited by a 5-HT_7_ antagonist or by dual inhibition (DDA and gallein). In this study, we suggest BJ-1113 being considered as a potential anticancer agent that suppresses TNBC progression, by inhibiting both the Gαs-linked generation of cAMP and activation of the PI3K/Akt pathway (Fig. 8), and thus, suppressing invasion and proliferation of MDA-MB-231 cancer cells stimulated by 5-HT or serum. The efficacy of BJ-1113 (at 10 μM) at suppressing 5-HT-induced cancer cell invasion was similar to that achieved by co-treating DDA (100 μM) and gallein (10 μM), which hints that BJ-1113 may target the autocrine action of 5-HT by inhibiting Gα and Gβγ signaling. However, because the inhibitory effect of BJ-1113 on cAMP production was not specific to G_s_ or G_i_ (Fig. 6b), BJ-1113 may not act at the 5-HT_7_ receptor itself. Rather, it inhibits adenylyl cyclase directly (Fig. 8). In addition, BJ-1113 did not inhibit phosphorylation of Src and ERK, signaling molecules downstream of Gβγ subunit. Therefore, inhibition of PI3K/Akt phosphorylation by BJ-1113 indicates that BJ-1113 may directly inhibit PI3K activity. This additional PI3K/Akt inhibitory activity of BJ-1113 could give rise to a stronger inhibitory effect in tumor growth than SB269970.

In addition to stimulating 5-HT production, we also demonstrate for the first time that autocrine 5-HT in MDA-MB-231 cells also stimulates the induction of VEGF, a well-known angiogenic factor that is produced and released by cancer cells. Furthermore, VEGF induction by 5-HT is mediated by the PI3K/Akt pathway, the same signaling pathway that increased cell proliferation in MDA-MB-231 cells and that was blocked by BJ-1113. Given the strong antiangiogenic activity of BJ-1113 against VEGF [30], the inhibitory effects of BJ-1113 on the expressions of TPH1 and VEGF in MDA-MB-231 cells indicate BJ-1113 exhibits the properties required of an anti-cancer agent for tumors resistant to anti-VEGF therapy. Moreover, considering that cancer is often accompanied by thromboembolic complications [36–38], significant levels of 5-HT released by activated platelets promote angiogenesis in endothelial cells, and thus, BJ-1113 could be expected to serve as a multi-faceted anti-angiogenic anti-cancer agent by acting on cancer cells and endothelial cells.

In the current study, in vivo anti-cancer activity of BJ-1113 was evaluated in a subcutaneous (s.c.) xenograft mouse model. The s.c. xenograft model does not provide tumor microenvironment interactions that lead to development of distant metastasis as in orthotopic xenografts. However, s.c. xenografts are easy to implant and measure the tumor size. Although future study evaluating in vivo efficacy of BJ-1113 in patient-derived tumor xenografts, which retain genetic characteristics and complex tumor heterogeneity evident in donor tumor from patients, is required before moving into clinical trial in drug development processes, our results showing strong anti-cancer activity of BJ-1113 without altering body weight in the s.c xenograft mouse model of MDA-MB-231 human breast cancer supports the promising possibility of BJ-1113 as an anti-cancer therapeutic agent.

## Conclusions

In summary, the current study demonstrates for the first time that autocrine 5-HT acts as a stimulator of breast cancer progression (Fig. 8). Considering the high expression of 5-HT and 5-HT_7_ receptors in TNBC and 5-HT-induced angiogenesis, we believe the suppression of 5-HT signaling linked to breast cancer progression and endothelial responses by using BJ-1113 constitutes a novel approach to cancer therapy against drug-resistant breast cancer growth and metastasis.

## Abbreviations

5-HT: 5-Hydroxytryptamine
ANOVA: Analysis of variance
BLI: Bioluminescent imaging
CAM: Chick chorioallantoic membrane
DDA: 2′,2′-dideoxyadenosine
ER: Estrogen receptor
FBS: Fetal bovine serum
HER-2: Human epidermal growth factor receptor 2
MAPK: Mitogen-activated protein kinase
PR: Progesterone receptor
qRT-PCR: Quantitative real-time polymerase chain reaction
RIPA: Radioimmunoprecipitation assay
TNBC: Triple-negative breast cancer
TPH1: Tryptophan hydroxylase 1
VEGF: Vascular endothelial growth factor
